# GCTOF-MS Combined LC-QTRAP-MS/MS Reveals Metabolic Difference Between Osteoarthritis and Osteoporotic Osteoarthritis and the Intervention Effect of Erxian Decoction

**DOI:** 10.3389/fendo.2022.905507

**Published:** 2022-07-28

**Authors:** Zhenyuan Ma, Yibao Wei, Li Zhang, Xiaoqing Shi, Runlin Xing, Taiyang Liao, Nan Yang, Xiaochen Li, Lishi Jie, Peimin Wang

**Affiliations:** ^1^Department of Orthopedics, Affiliated Hospital of Nanjing University of Chinese Medicine, Nanjing, China; ^2^Key Laboratory for Metabolic Diseases in Chinese Medicine, First College of Clinical Medicine, Nanjing University of Chinese Medicine, Nanjing, China; ^3^Department of Traditional Chinese Medicine Orthopedics, Jiangsu Province Hospital of Chinese Medicine, Nanjing, China

**Keywords:** knee osteoarthritis, osteoporosis, metabolomics, erxian decoction, osteoporotic osteoarthritis

## Abstract

**Purpose:**

OP and OA are chronic bone diseases with high incidence in the middle-aged and elderly populations. The latest research shows that the pathological environment of OP may be involved in the aggravation of the pathological process of OA, and the pathological state of OP plays an important role in the aggravation of OA pathology. EXD is a traditional Chinese medicine decoction that has been used to treat osteoporosis. Therefore, we further study whether OA will be aggravated in the OP environment and whether EXD can alleviate OA by intervening in the OP environment. The purpose of this study was to analyze the effect of OP on OA metabolites by using metabolomic methods and to explore the intervention mechanism of EXD on osteoporotic OA.

**Method:**

Thirty-two SD rats were randomly divided into normal group, OA group, OP-OA group, and EXD group. EXD was administered by gavage. Histopathological evaluation of cartilage tissue was performed using Saffron fast green and HE staining. Western blot and qRT-PCR were used to detect the expression levels of chondrogenesis genes SOX9, COL2A1, and COMP in cartilage tissue. GC-TOFMS and LC-QTRAP-MS/MS metabolomics methods were used to analyze the changes of metabolites in serum samples of rats in each group.

**Result:**

The slice results showed that the cartilage damage in the OP-OA group was more serious than that in the OA group, which was significantly relieved after EXD intervention, indicating that the cartilage damage in the OP-OA group was more severe than that in the OA group and further reduced the protein and gene expressions of cartilage markers SOX9, COL2A1, and COMP. Thirty-seven substances were identified, and gentiopicroside, emodin, quercetin, and diosmetin were analyzed as possible active components of EXD. EXD treatment significantly reduced cartilage damage and reversed the expression of these markers. Metabolomics showed that EXD attenuated cartilage destruction by modulating the expression of cystine, chenodeoxycholate, and D-Turanose, involving glycolysis/gluconeogenesis, pantothenate, and CoA biosynthesis metabolic pathways.

**Conclusion:**

The OP environment may promote the progression of OA through metabolic factors. The benign intervention of EXD in osteoporotic OA involves cystine, chenodeoxycholate, and D-Turanose, and their associated glycolysis/gluconeogenesis, pantothenate, and CoA biosynthesis metabolic pathways. Therefore, we have a deep understanding of the metabolic-related intervention of EXD in osteoporotic OA and are eager to better understand the mechanism of multi-targeted intervention of EXD in bone metabolic lesions.

## Introduction

Osteoporosis (OP) and osteoarthritis (OA) are the two most common diseases in the chronic degeneration of the musculoskeletal muscle system. The incidence rate is increasing year by year, which seriously endangers the physical and mental health of human beings (1–4). Previous studies have shown that metabolic disorders are the main pathological features of OP, manifested as osteopenia, fracture of trabecular bone, and thinning of cortical bone (5), while the main pathological features of OA are cartilage loss, osteophyte formation, and synovial inflammation in the trochlear joint (6). Therefore, OP has been considered to be inversely associated with OA for a long time, and low bone mass may even have a protective effect on OA at that time.

With the deepening of research, scholars have gradually realized that OP and OA may also occur in the same patient. Chu L et al. show that there are similarities in the pathological changes of subchondral bone in OP and OA patients. More ideas in recent years have suggested that SB loss may negatively affect osteochondral biomechanics, ultimately accelerating OA progression to osteoporotic OA (7). Different from the characteristic subchondral bone sclerosis and hyperplasia of OA, the disorder of subchondral bone metabolism in the early stage of OA is mainly bone resorption, showing decreased bone mass, decreased trabecular bone, and stress microfracture, which is similar to OP (6). At the same time, the above pathological changes tend to take precedence over cartilage degeneration in OA in time, indicating that the metabolic disorder of subchondral bone may be an important reason for the development of OA, and OP may have a driving effect on the progression of OA. Unfortunately, the specific effector targets and mechanism of action in this process are still lacking reports.

The reason why recent studies have focused on the role of metabolic abnormalities in Osteoporotic OA is precisely due to the similarities in the loss of bone homeostasis between OP and OA (8). Studies have shown that under high body mass index, excessive adipokines release and metabolic changes lead to the occurrence of OP and increased fracture risk (9–11). It is well known that a high body mass index not only brings more mechanical load to OA by increasing body weight but also increases the risk of OA by inducing sterile inflammation through adipokines (12). Therefore, the abnormal lipid metabolism accompanying the pathological progress of OP may induce OA at the same time, thereby accelerating the progress of OA. Metabolic differences in accelerated OA progression provide the possibility and a rationale for the treatment of Osteoporotic OA.

From a therapeutic point of view, some drugs for the treatment of OP, such as Pueraria lobata and Rhizoma Drynariae, etc., can also benefit OA in the clinical treatment of OA, that is, cutting off the progress of OP may alleviate OA (13–16). The traditional Chinese medicine decoction ErXian Decoction (EXD) is included in the famous medical book “Wenbing Tiaobian” in the Ming Dynasty (17). It consists of epimedium, curcuma, Morinda officinalis, angelica, phellodendron, and anemarrhena. It was widely used in the treatment of osteoporosis with a definite curative effect (18–21). Interestingly, EXD also achieved satisfactory results in the treatment of OA. Combined with the above, it is reasonable to speculate that the benefit of EXD in the treatment of OP and OA may be aimed at the metabolic disorders shared by the two diseases and may be used for the treatment of Osteoporotic OA. Therefore, in this study, an experimental animal OA model and Osteoporotic OA model were constructed by surgery, and gas chromatography-time of flight mass spectrometry (GC-TOFMS) and liquid chromatography quadrupole ion trap tandem mass spectrometry (LC-QTRAP-MS/MS) techniques were combined to study the metabolomic differences and related mechanisms under the action of OA, Osteoporotic OA and EXD.

## Material and Methods

### Preparation for EXD

The herbs that make up EXD include 9 g epimedium, 9 g curcuma, 9 g Morinda officinalis, 9 g angelica, 6 g phellodendron, 6 g anemarrhena (all herbs were obtained from Jiangsu Provincial Hospital of Traditional Chinese Medicine) (22), and all herbal materials used in our study were approved by Nanjing University of Traditional Chinese Medicine. It met the quality requirements of the 2015 edition of the Chinese Pharmacopoeia. Four times the mass of water for each sub-medicine were added, mixed and soaked, boiled two times, 30 min each time, and extracted three times.

### Experimental Design

Thirty-two 2-month-old Sprague-Dawley (SD) female rats used in the experiment were provided by Nanjing Qinglongshan Animal Farm (ethical number: ACU211204), weighing 180-220 g. Rats were maintained in a specific pathogen-free laminar flow environment at a temperature of 25 ± 2°C, humidity 55%, and a 12-h light/dark regimen. The animal care and use protocol followed the National Institutes of Health Guide for the Care and Use of Laboratory Animals and was approved by the Animal Care and Use Committee of Nanjing University of Chinese Medicine.

The rats were randomly divided into four groups: Normal group (n=8), OA group (n=8), Osteoporotic-osteoarthritis (OP-OA) group (n=8), and EXD group (n=8). The model of OA was obtained by amputating the anterior cruciate ligament of the rat knee joint by ACLT method, and the ACLT model was determined by the anterior drawer test (23). The OP model was obtained by removing both ovaries from rats using bilateral oophorectomy according to the references (24). In the OP-OA group and EXD group, both ACLT method and bilateral oophorectomy were used to establish the model. After 14 days of successful modeling, the EXD group was given EXD by intragastric administration at a dose of 0.5 ml/100 g for 21 consecutive days and other groups were used equal amounts of normal saline. After the last intragastric administration of EXD, blood was collected from the abdominal aorta and cartilage tissue was collected from the rats in each group.

### Sample Collection and Preparation

After the last intragastric administration of EXD, the rats were anesthetized by intraperitoneal injection of 3% pentobarbital, and blood was collected from the abdominal aorta. The rats in each group were sacrificed and serum and cartilage tissues were collected. The rat’s knee joint was shaved, and the skin was incised along both sides of the patellar ligament. From the upper edge of the patella, the distal quadriceps muscle was incised laterally to the femur, and the free patella and its surrounding tissues were pulled up and opened to the distal end. Exposing the knee joint, the surrounding tissue and the anterior and posterior cruciate ligaments were cut off. Exposing the tibial plateau, the cartilage was carefully cut to maintain its integrity as much as possible. Cartilage tissues from two rats were randomly selected from each group and stored in 4% paraformaldehyde for pathological sectioning.

### Micro-CT Examination

Rats were randomly selected from each group and the hip joint was removed. The intact tissue of the knee joint was preserved and stored in 4% paraformaldehyde for Micro CT examination. Imaging system (model Quantum GX, USA).

### Histopathological Analysis

The cartilage tissue was fixed with 4% paraformaldehyde, soaked in EDTA, embedded in paraffin, and sectioned for hematoxylin-eosin (HE) staining.

The tissue sections were gradient dehydrated, and then stained using safranin O-fast green cartilage staining kit according to the instructions.

From the cartilage structure, chondrocyte density, and inflammatory cell infiltration degree, Mankin’s score was used to detect the degree of damage to the knee articular cartilage tissue in each group of rats (25). Scores were 0-1 no cartilage destruction, 2-4 low-grade cartilage destruction, and 5-9 high-grade cartilage destruction. The standard is shown in **Table 1**.

**Table 1:** Markin’s Score standard (25).

Classification	Feature	Score
Cartilage structure	Normal	0
	Surface irregularities	1
	Mild reduction in cartilage thickness	2
	Moderate reduction in cartilage thickness	3
	Severe reduction in cartilage thickness	4
Cartilage cells	Normal	0
	Slight increase	1
	Moderate increase	2
	Severe increase	3
Inflammatory infiltrate	Normal	0
	Slight increase	1
	Moderate increase	2
	Severe increase	3

### Western Blotting

The cartilage tissue of each group of rats was cut into pieces, poured into liquid nitrogen for 10 min, and then RIPA lysis solution containing 0.1% PMSF was added to mix for 10 min. After standing on ice for 30 min, the supernatant was collected after centrifugation. Protein levels were quantified using a BCA protein detection kit (Beyotime Biotechnology, Shanghai, China). The protein was denatured at 98°C for 7 min. The proteins were separated by electrophoresis, transferred from the gel to PVDF membrane, and blocked with 5% nonfat milk for 2 h. The membrane was incubated with the COL2A1, SOX9, β-actin, COMP overnight at 4°C, and then incubated with the secondary antibody (1:5000 affinity, USA) at room temperature for 2 h, and then the bands were exposed by ECL. Gray value was quantified with β-actin as an internal marker, i.e., target protein gray value/internal reference overall gray value.

### Quantitative Real-Time Polymerase Chain Reaction

The RNA of cartilage tissue was extracted with Trizol solution, and the purity and concentration of RNA were determined by spectrophotometer. Reverse transcription was performed using 5 × HiScript II qRT Super Mix according to the kit instructions (vazyme, Nanjing, China). qRT-PCR was performed using a SYBR Premix Ex Taq II according to the manufacturer’s instructions in an ABI PRISM 7300 (Applied Biosystems, USA) instrument. Primers were designed and synthesized by Shanghai Biotechnology Service Company. The primer sequences are as follows:

**Table:** 

Gene	Forward Primer	Reverse Primer
COL2A1	5′-GGAGCAGCAAGAGCAAGGAGAAG-3′	5′-TCAGTGGACAGTAGACGGAGGAAAG-3′
SOX9	5′-GGGCTCTACTCCACCTTCACCTAC-3′	5′-GCTGTGTGTAGACGGGTTGTTCC-3′
COMP	5′-GGGTGGTGCTCAATCAGGGAATG-3′	5′-GAAGCCAGCGTAGTCATCATCGG-3′
β-actin	5′-GAGAGGGAAATCGTGCGT-3′	5′-GGAGGAAGAGGATGCGG-3′

The mRNA levels of each gene were normalized to β-actin and calculated using the 2^-ΔΔ^CT data analysis method.

### Processing and Testing of EXD

After thawing on ice, the EXD samples were vortexed for 30 s, centrifuged for 15 min at 4°C, 12000 rpm (centrifugal force 13800 ×g, radius 8.6 cm), 300 μL of supernatant was taken into a 1.5 ml EP tube, add 1000 μL Extraction solution (methanol:water = 4:1, internal standard concentration of 10 μg/mL), vortexed for 30 s, sonicated in ice-water bath for 5 min, after standing at -40°C for 1 h, the sample was placed at 4°C, 12000 rpm (centrifugal force 13800 ×g, radius 8.6 cm), and centrifuged for 15 min, the supernatant was taken out and passed through a 0.22 μm filter membrane, injected into the sample bottle, and tested ion the machine. Then 100 μL of each sample was mixed to form a QC sample.

LC-MS/MS analysis was performed on an UHPLC system (Vanquish, Thermo Fisher Scientific). The flow rate was set at 0.5 mL/min and the sample injection volume was set at 5 μL. The mobile phase consisted of 0.1% formic acid in water (A) and 0.1% formic acid in acetonitrile (B). The multi-step linear elution gradient program was as follows: 0-11 min, 85-25% A, 11-12 min, 25-2% A, 12 -14 min, 2-2% A, 14-14.1 min, 2-85% A, 14.1-16 min, 85%-85% A.

An Orbitrap Exploris 120 mass spectrometer coupled with an Xcalibur software was employed to obtain the MS and MS/MS data based on the IDA acquisition mode. Ion Transfer Tube Temp: 350°C, Vaporizer Temp: 350°C, Sheath gas flow rate: 35 Arb, Aux gas flow rate: 15 Arb, Full ms resolution: 60000, MS/MS resolution: 15000, Collision energy: 16/38/42 in NCE mode, Spray Voltage: 5.5 kV (positive) or-4 kV (negative).

### Analysis Conditions of GC/LC-MS Treatment of the Serum Samples of Rats

After the sample serum was thawed at 4°C, 50 μL of the sample was accurately weighed into a 2 mL EP tube, and 1 mL of a mixed solution of isopropanol: acetonitrile: water (3:3:2, v/v/v) was added and sonicated at room temperature for 5 min. Then centrifuge at 14,000 rpm for 2 min, 500 μL of the supernatant taken and added to a new 2 mL EP tube (remaining backup), and the supernatant placed in a vacuum concentrator to concentrate to dryness. GC-MS analysis: added 80 μL of 20 mg/mL methoxyamine solution to the evaporated sample, vortexed for 30 s, and incubated at 60°C for 60 min, then added 100 μL of BSTFA reagent, and incubated at 70°C for 90 min, centrifuge at 14,000 rpm for 3 min, 90-100 μL of the supernatant taken and added to the liner in the GCMS detection bottle, and then 20 μL taken from each sample to be tested and mixed into a QC sample. LC-MS analysis: added 200 μL of acetonitrile:methanol (1:1) to the thoroughly evaporated sample, vortexed for 30 s, centrifuged at 14,000 rpm for 3 min, 90-100 μL of the supernatant added to the LCMS detection bottle Then 20 μL of each sample to be tested taken and mixed into a QC sample.

GC-MS conditions: Agilent 7890 gas chromatography time of flight mass spectrometer is used for detection. The specific analysis conditions of GC-TOF-MS are as follows: Column: DB-5MS(30m×250μm×0.25μm), Sample Volume: 1μL, Column Flow:1mL min−1, Front Injection Temperature: 280°C, Transfer Line Temperature: 320°C, Ion Source Temperature: 230°C, Electron Energy: 70eV, Mass Range:m/z: 75-650, Acquisition Rate: 10 spectra per second. Ms-dial software was used to analyze the mass spectrometry data, such as peak extraction, peak integration, peak alignment, baseline correction, and deconvolution (17). Fiehnbinbase database (18) was used for substance qualitative work, including retention time index matching and mass spectrometry matching. Finally, the peaks were removed with the detection rate ≤ 50% in QC samples or the detection rate < 50% or RSD > 30% in each group except the QC group (19).

LC-MS conditions: Chromatographic conditions: The samples were separated using a 7890b high-performance liquid chromatograph (Agilent, USA) and equipped with a CSH C18 column (1.7 μm,2.1 × 100mm). Injection volume: 2μL, Flow rate: 0.30 L/min, Mobile phase A: water (containing 0.1% FA), Mobile phase B: acetonitrile, the gradient elution procedure is shown as follows:

**Table:** 

Time(min)	A	B
0	95%	5%
1	95%	5%
11	1%	99%
13	1%	99%
13.1	95%	5%
15	95%	5%

The column temperature was 65°C, and the injection volume was 2 μL in both positive and negative ion mode. Mass spectrum conditions: Pegasus BT gas chromatography time of flight mass spectrometer (LECO, USA) was used to detect samples. The instrument parameters are as follows: Scanning mode: SMRM IDA EPI, Curtain Gas (CUR): 35psi, Collision Gas (CAD): Medium, Temperature (TEM): 550°C, Ion Source Gas 1 (GS1): 60psi, Ion Source Gas 2 (GS2): 60psi, Ion Spray Voltage (IS): 5500V (Positive ion mode)/-4500V (Negative ion mode).

### Statistical Analysis

Mass spectral data were analyzed by peak extraction, peak integration, and peak alignment using ABOX software. *P<0.05* indicates statistical significance. The raw data were converted to Abf format using Abf Converter software (http://www.reifycs.com/AbfConverter/), and positive and negative ion mode data were imported into MS-DIAL software for data preprocessing, filtering, alignment, and peak identification. Identified metabolites, retention times (Rt), mass-to-charge ratios (m/z), and peak information was obtained for all samples. The data exported from MS-DIAL was normalized by the SERRF algorithm through the R language. The fold change (FC) was compared with the median peak height of each group using the R language. FC>2.0 was considered to indicate a significant difference. Principal component analysis and cluster analysis were performed on the data using Metaboanalyst (http://www.metaboanalyst.ca/). Analysis was performed using GraphPad Prism software 8.0 (Solvusoft Corporation, USA). Statistical analysis was performed using SPSS 20.0 software (SPSS Inc., Chicago, IL, USA). Data are presented as mean ± standard deviation. Group comparisons were assessed with one-way ANOVA. A value of *P<0.05* was considered statistically significant. Quantitative data were expressed as mean plus or minus standard deviation, and normal distribution was tested by Student’s t-test.

## Result

### EXD Can Relieve Cartilage Destruction of Osteoporotic Osteoarthritis and Promote Cartilage Repair

**Figure 1:**
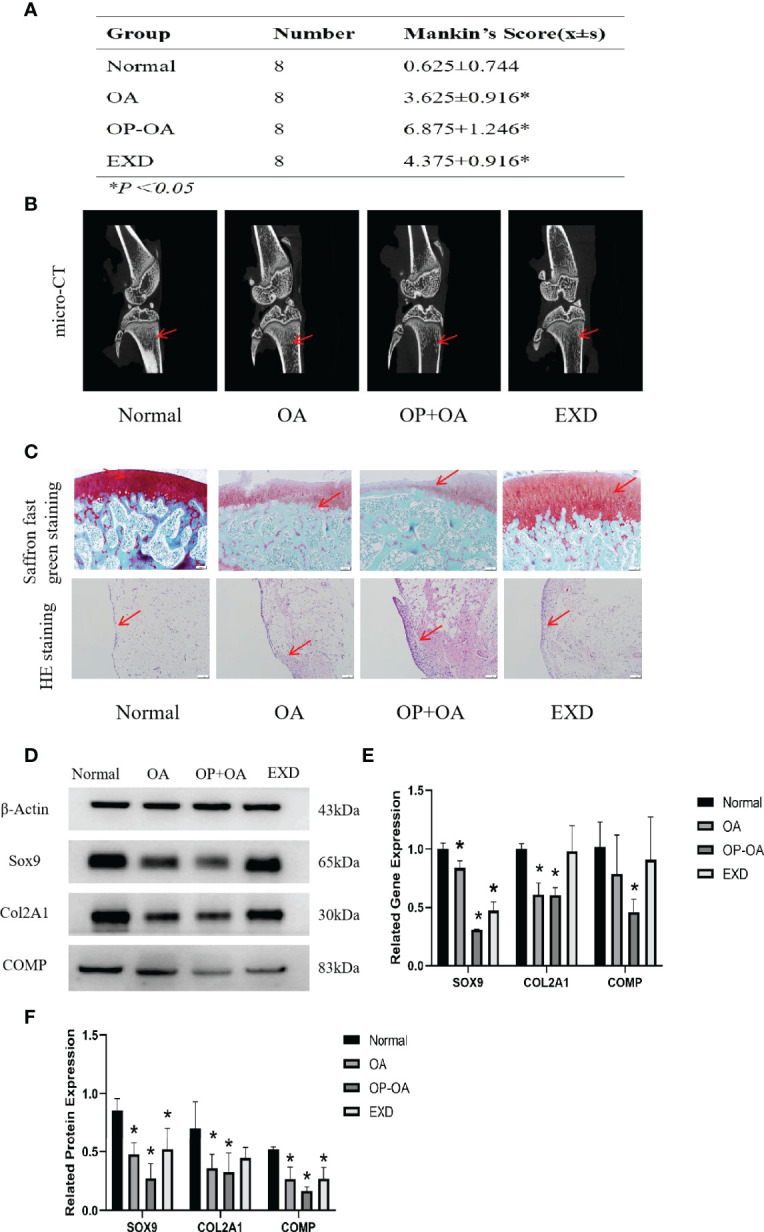
EXD can relieve cartilage destruction of Osteoporotic OA and promote cartilage repair. **(A)** The Mankin’s score of the normal group and EXD group were significantly lower than those of the OA group and OP-OA group. The score of OP-OA group was higher than that of OA group, and the inflammatory infiltration and cartilage damage were obvious. **(B)** Micro-CT verification of osteoporotic trabecular bone in each group of rats. Compared with normal and OA, the trabecular bone in the proximal tibia of OP-OA was sparse and showed low signal density, while the trabecular bone in the proximal tibia after EXD intervention was dense and showed high signal density. The red-pointing area indicates the increase or decrease of trabecular bone. **(C)** Cartilage histomorphology of each group stained with safranin O-fast green and H&E,100x scale bar=100 μm. Red-pointing areas indicate cartilage changes and cellular infiltration. **(D, E)** Western blot analysis of the effects of EXD on SOX9, COL2A1, and COMP in rat cartilage tissue. **(F)** Relative protein and gene expression of SOX9, COL2A1, and COMP in each group of cartilage tissue. *P<0.05.

In order to explore the intervention effect of EXD on Osteoporotic OA, safranin O-fast green and HE staining were performed on cartilage tissue of rats in each group, and Mankin’s score was calculated. Micro-CT was used to identify osteoporosis in each group of rats, and gene and protein expressions were analyzed by PCR and WB. **Figure 1A** shows that the Mankin’s score of the OA group and the OP-OA group were significantly higher than those of the normal group, the score of the OP-OA group was higher than the OA group, and the score of the EXD group was significantly lower. **Figure 1B** shows that the OP-OA group had more severe osteoporosis than the OA group and the normal group. Compared with OA group, the trabecular bone was sparse and low in signal density in the proximal tibia region of the OP-OA group, and this change was improved after EXD intervention. **Figure 1C** shows that compared with the normal group, the OP-OA group and OA group have more inflammatory cell infiltration in HE-stained sections, and OP-OA group has more inflammatory cell infiltration than OA group, and inflammatory cells after EXD intervention infiltration was relieved. At the same time, under the Saffron fast green staining section, it was found that compared with the normal group, the cartilage damage of the OP-OA group and OA group was obvious, and the cartilage damage of the OP-OA group was more serious, which was alleviated after EXD intervention. **Figures 1D, E** show that the degree of cartilage degeneration was increased in the OP-OA group compared with the OA group due to decreased protein expression of COL2A1, SOX9, and COMP. Cartilage degeneration was improved under EXD intervention due to elevated COL2A1, SOX9, COMP protein expression. **Figure 1F** shows that the degree of cartilage degeneration was increased in the OP-OA group compared with the OA group due to decreased gene expression of COL2A1, SOX9, and COMP. Cartilage degeneration was improved under EXD intervention due to elevated COL2A1, SOX9, and COMP gene expression.

### EXD Active Substance Analysis

**Figure 2:**
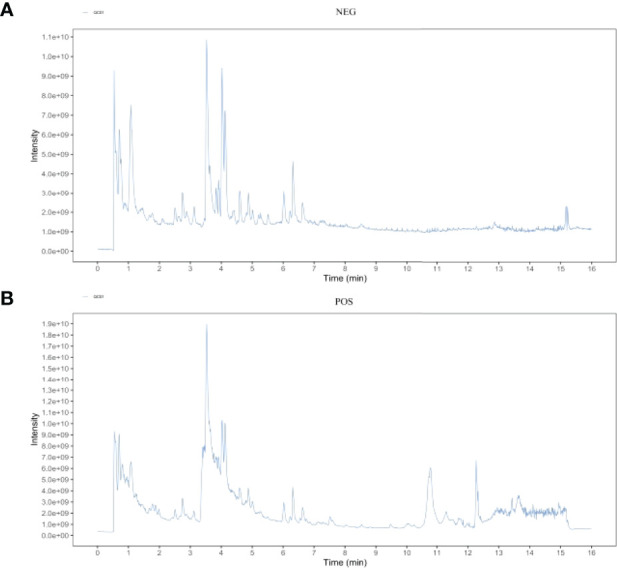
Total ion chromatogram of the methanol extract acquired by UHPLC-QE-MS in the positive **(A)** and negative **(B)** ion modes.

The main components of the compounds in the EXD soup were preliminarily identified using UHPLC-QE-MS omics detection. **Figures 2A, B** depict the base peak chromatogram of EXD extract. The chemical components identified in the EXD water extract (**Tables 2**, **3**) describe 12 compounds in negative ion mode and 25 compounds in positive ion mode obtained by analysis (**Table 3** is the positive and **Table 2** is negative ion modes). We identified compounds such as Gentiopicroside, emodin, Quercetin, etc.

**Table 2:** NEG.

NO.	Name	Formula	m/z	RT(min)	Mass Error
1	osmanthuside H	C19H28O11	431.1551526	0.64	-0.910791742
2	p-Hydroxybenzaldehyde	C7H6O2	485.1794385	0.96	-0.524950437
3	Gentiopicrin	C16H20O9	563.1361209	1.11	-0.813226144
4	Phellodendrine chloride	C20H24NO4+.Cl-	303.0504314	1.23	-0.782729256
5	Vicenin III	C26H28O14	221.0813696	1.35	-0.856395644
6	Luteolin-4’-O-glucoside	C21H20O11	447.0927647	1.68	-0.774604863
7	Rutaevin	C26H30O9	465.1382636	3.44	-0.574260606
8	Syringetin-3-O-glucoside	C23H24O13	255.2325419	4.35	-0.485006134
9	Eupatilin	C18H16O7	1063.534338	4.70	-0.178689562
10	Emodin	C15H10O5	787.2661516	5.43	0.209983782
11	limonin	C26H30O8	821.3932051	5.52	-0.293723641
12	(+)-Pinoresinol	C20H22O6	493.22818	5.98	0.004940317

**Table 3:** POS.

NO.	Name	Formula	m/z	RT(min)	Mass Error
1	Guanine	C5H5N5O	298.27411	0.57	-0.775697869
2	Anisic aldehyde	C8H8O2	369.1178286	0.76	-0.665764759
3	picein	C14H18O7	405.2619891	1.29	-0.758662736
4	beta-Asarone	C12H16O3	169.0496061	1.32	-0.621750573
5	methyl chlorogenate	C17H20O9	266.1729328	1.39	-0.773373586
6	p-Coumaric acid	C9H8O3	531.186223	1.79	-0.349277895
7	cirsimaritin	C17H14O6	455.1532487	1.81	-0.678218407
8	Quercetin	C15H10O7	240.2323075	1.83	-0.63710176
9	Triptonide	C20H22O6	287.0908944	2.79	-0.534481958
10	Ethyl 4-methoxycinnamate	C12H14O3	147.0441382	3.01	-0.126874874
11	Phellodendrine chloride	C20H24NO4+.Cl-	123.0440145	3.21	-0.437036096
12	Isoeugenol acetate	C12H14O3	132.1018832	3.25	-0.057361005
13	Scutellarein	C15H10O6	127.0389746	3.29	-0.313094459
14	Isoflavone base + 3O	C15H10O5	251.1641206	3.99	-0.115898914
15	18 beta-Glycyrrhetintic Acid	C30H46O4	153.0544604	4.50	-0.427811004
16	wogonin	C16H12O5	265.1434982	5.21	0.097311555
17	trans-pterostilbene	C16H16O3	166.1226703	5.77	0.346799361
18	aschantin	C22H24O7	905.4710905	6.13	-0.119874283
19	Isoflavone base + 3O	C16H12O6	104.1069657	6.26	0.247654695
20	aflatoxin B1	C17H12O6	355.1179751	7.21	0.381589661
21	Periplogenin	C23H34O5	333.2041695	9.26	0.420508951
22	Glyceryl linolenate	C21H36O4	287.1272354	10.73	0.714953565
23	Acetophenone	C8H8O	233.1534317	10.73	4.318311117
24	Chaulmoogric Acid	C18H32O2	781.4359698	12.29	1.472971925
25	Curcumenol	C15H22O2	365.1010515	12.92	2.015139985

### Metabolomics Reveals Relevant Differential Metabolites Between OA & OP-OA

**Figure 3:**
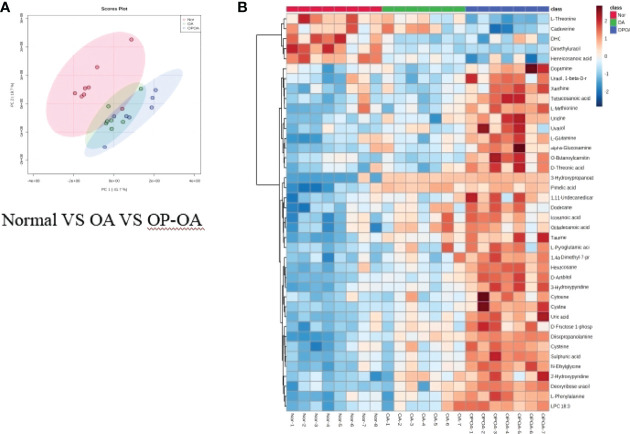
Metabolomics reveals relevant differential metabolites between normal, OA, & OP-OA. **(A)** PCA chart of the normal group, OA group, and OP-OA group. **(B)** Comparison heat map of differential metabolites in the normal group, OA group, and OP-OA group.

Principal component analysis was performed on serum metabolites in the normal group, OA group, and OP-OA group. The results are shown in **Figure 3A**. Each point in the graph represents a sample, and all sample points are distributed within a circular area (95% confidence interval). The metabolites between the normal group and the OA group were significantly different, and the metabolites in the OP-OA group and the OA group were separated and kept away from the normal group, suggesting that the OP environment further disturbed the levels of metabolites in the rat and aggravated the progression of OA.

Compared with the normal group, 10 differential metabolites were obtained in the OA group compared with the normal group with a Fold change >2.0, one showed an upward trend and nine showed a downward trend (**Table 4**). Under the same standard, the OP-OA group obtained 15 differential metabolites compared with the OA group, four showed an upward trend, and 11 showed a downward trend (**Table 5**). The data for these differential metabolites were analyzed to obtain the heatmap shown in **Figure 3B**. Each square in the figure represents the mean value of the corresponding intensity of the metabolites in each group of samples, with red representing an increase in intensity and blue representing a decrease in intensity. The changes of some metabolites in the OP-OA group and the OA group were obvious, and there was a significant difference with the normal group (*P<0.05*). Therefore, it is speculated that the OP environment affects the levels of these metabolites such as Cystine, Chenodeoxycholate, D-mannitol, (S)-oleuropein, etc. and related metabolic pathways, leading to the further development of OA.

**Table 4:** Differential metabolites.

Metabolite name	Fold Change	log2 (FC)
PE 36:2	0.32360	-1.6277
N-Amidino-L-aspartate	0.32877	-1.6048
LPA 20:1	0.35527	-1.493
Lactulose	0.38990	-1.3588
D-Mannitol	0.39721	-1.332
Taurine	0.41287	-1.2762
D-Turanose	2.26160	1.1773
(S)-Oleuropeic acid	0.45201	-1.1456
3-Hydroxypropanoate	0.45242	-1.1443
Cystine	0.49750	-1.0072

**Table 5:** Differential metabolites.

Metabolite name	Fold Change	log2(FC)
Isophthalic acid	0.10410	-3.264
3-Epicholic acid	0.26873	-1.8958
(S)-Oleuropeic acid	3.34890	1.7437
Hexanal	3.30350	1.724
Cholic Acid	0.35652	-1.4879
Chenodeoxycholate	0.39922	-1.3248
Cystine	0.41314	-1.2753
D-Mannitol	2.34360	1.2288
O-Butanoylcarnitine	0.42678	-1.2284
Lithocholic acid	0.47054	-1.0876
Lactulose	2.10370	1.0729
Acar 9:1	0.47690	-1.0682
PHE-AC-GLN-OH	0.48971	-1.03
D-Lyxose	0.49826	-1.005
alpha-Glucosamine 1-phosphate	0.49856	-1.0042

### Metabolomics reveals correlated differential metabolic pathways between normal, OA, and OP-OA

**Figure 4:**
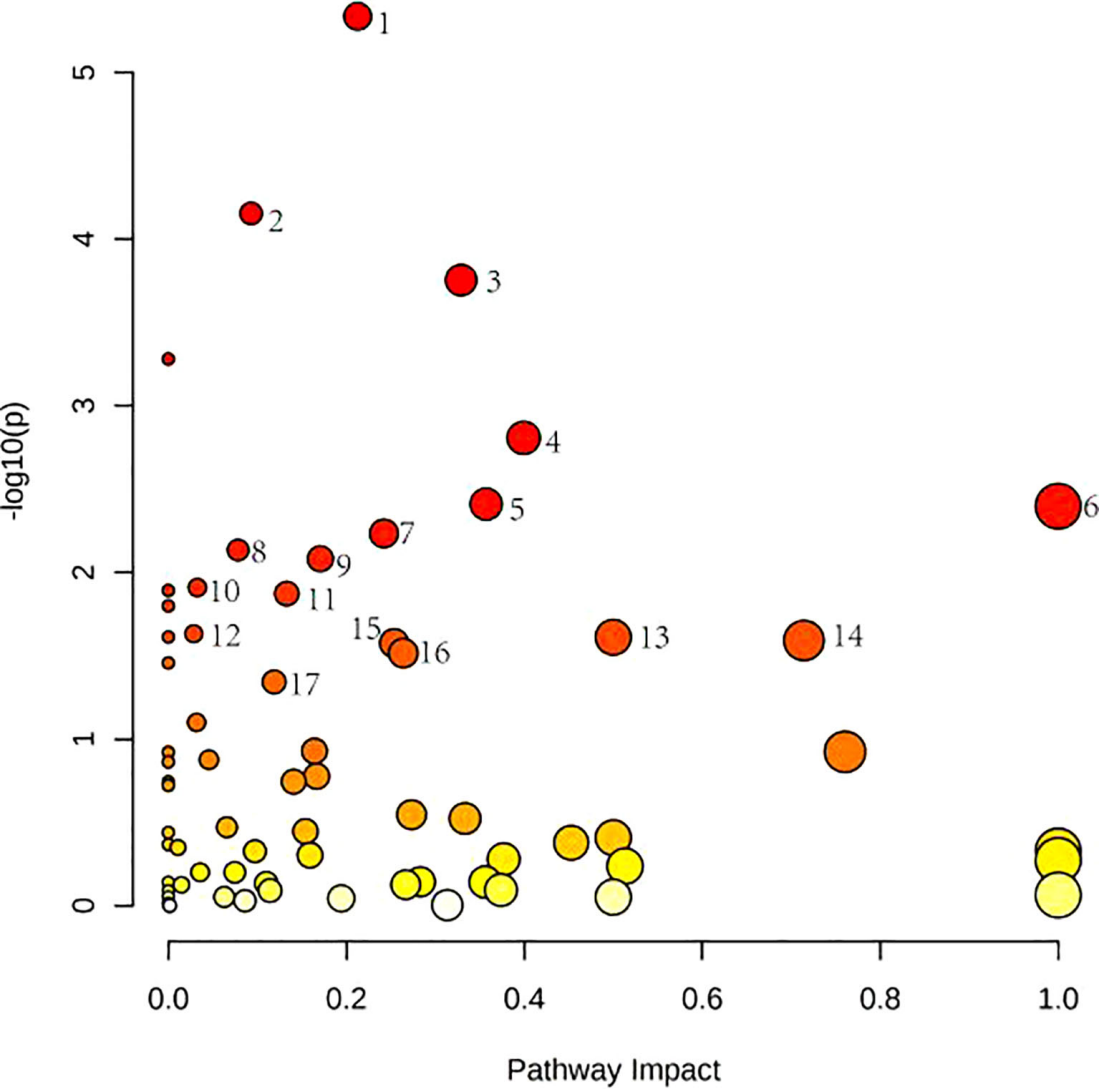
Metabolomics reveals correlated differential metabolic pathways between normal, OA, and OP-OA. Metaboanalyst (http://www.Metabo-analyst.ca/) carried out metabolic pathway enrichment analysis, with P<0.05; 17 related metabolic pathways were analyzed.

**Figure 4** shows these differential metabolites were further altered in the OP-OA group were imported into Metaboanalyst (http://www.Metabo-analyst.ca/) for metabolic pathway enrichment analysis, and 17 related metabolites were obtained by *P<0.05* analysis pathways (**Table 6**): Sulfur metabolism, purine metabolism, pyrimidine metabolism, beta-Alanine metabolism, phenylalanine metabolism, tyrosine and tryptophan biosynthesis, retinol metabolism, vitamin B6 metabolism, pentose phosphate pathway, steroid hormone biosynthesis, steroid biosynthesis, pantothenate and CoA biosynthesis, D-glutamine and D-glutamate metabolism, taurine and hypotaurine metabolism, arginine biosynthesis, cysteine ​and methionine metabolism, and glutathione. The comparison results show that under the intervention of OP, the most significant effect was sulfur metabolism.

**Table 6:** Pathway.

NO.	Pathway Name	Match Status	p	-log(p)	FDR	Impact	Details
1	Sulfur metabolism	1/8	4.61E-06	5.3365	3.09E-04	0.21277	KEGG SMP
2	Purine metabolism	9/65	7.01E-05	4.154	0.00235	0.09299	KEGG SMP
3	Pyrimidine metabolism	10/39	1.76E-04	3.7544	0.00393	0.32899	KEGG SMP
4	beta-Alanine metabolism	4/21	0.00156	2.8076	0.02087	0.39925	KEGG SMP
5	Phenylalanine metabolism	3/10	0.00389	2.4104	0.03831	0.35714	KEGG SMP
6	Phenylalanine, tyrosine and tryptophan biosynthesis	3/4	0.00400	2.3977	0.03831	1	KEGG SMP
7	Retinol metabolism	1/17	0.00583	2.2345	0.04881	0.24227	KEGG SMP
8	Vitamin B6 metabolism	1/9	0.00733	2.1348	0.05458	0.07843	KEGG SMP
9	Pentose phosphate pathway	2/22	0.00828	2.0822	0.05545	0.17086	KEGG SMP
10	Steroid hormone biosynthesis	4/85	0.01232	1.9096	0.06900	0.03261	KEGG SMP
11	Steroid biosynthesis	3/42	0.01339	1.8733	0.06900	0.13308	KEGG
12	Pantothenate and CoA biosynthesis	6/19	0.02330	1.6326	0.09357	0.02857	KEGG SMP
13	D-Glutamine and D-glutamate metabolism	3/6	0.02445	1.6118	0.09357	0.5	KEGG SMP
14	Taurine and hypotaurine metabolism	3/8	0.02558	1.592	0.09357	0.71428	KEGG SMP
15	Arginine biosynthesis	8/14	0.02654	1.5762	0.09357	0.2538	KEGG
16	Cysteine and methionine metabolism	5/33	0.03046	1.5163	0.10203	0.26401	KEGG SMP
17	Glutathione metabolism	6/28	0.04534	1.3435	0.13809	0.11891	KEGG SMP

### Metabolomics Reveal Relevant Differential Metabolites Between Normal, OP-OA, and EXD

**Figure 5:**
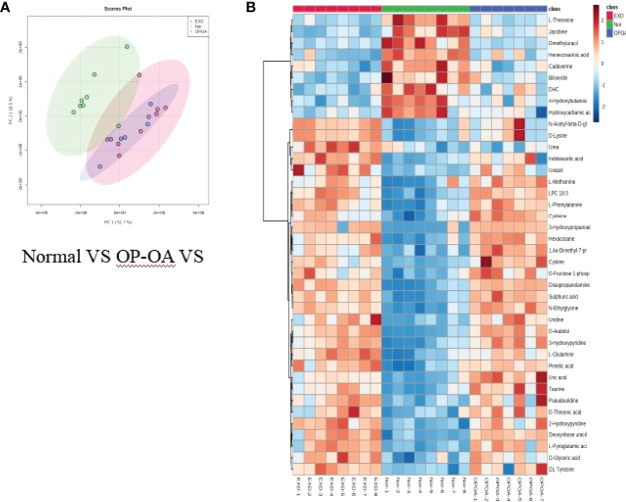
Metabolomics reveals relevant differential metabolites between normal, OP-OA, and EXD. **(A)** PCA chart of the normal group, OP-OA group, and EXD group. **(B)** Comparison heat map of differential metabolites in the normal group, OP-OA group, and EXD group.

Principal component analysis was performed on the metabolites of the normal group, OP-OA group, and EXD group, and the results are shown in **Figure 5A**. Each point in the figure represents a sample, and all sample points are distributed in a circular area (i.e., 95% confidence interval). The metabolites in the OP-OA group and the normal group were separated, suggesting that the OP environment further disturbed the levels of metabolites in rats, thereby further aggravating the destruction of OA cartilage, but it was visible after EXD intervention. The EXD group was significantly different from the OP-OA group, and migrated to the normal group, suggesting that EXD could regulate the metabolites in rats to normal levels, thereby reducing cartilage damage. The metabolites obtained in the normal group and the OP-OA group were processed and analyzed respectively, and 27 differential metabolites were obtained with a fold change >2.0 as the standard, of which four showed an upward trend and 23 showed a downward trend (**Table 7**). Fourteen differential metabolites were observed between the OP-OA group and the EXD group, and nine showed an upward trend and five showed a downward trend (**Table 8**). The normalized data of the differential metabolites in the OP-OA group and the EXD group were subjected to univariate analysis, and a heat map was made as shown in **Figure 5B**. The concentrations of some metabolites in the EXD group have significant changes compared with the OP-OA group, and there was a significant difference compared with the normal group (*P<0.05*). Therefore, it is speculated that EXD may play a role in promoting OP-OA cartilage repair by affecting the levels of isophthalic acid, D-Turanose, (S)-Oleuropeic acid, and chenodeoxycholate metabolites and related metabolic pathways.

**Table 7:** Differential metabolites.

Metabolite name	Fold Change	log2(FC)
Isophthalic acid	0.15913	-2.6517
Cystine	0.20554	-2.2825
N-Amidino-L-aspartate	0.26904	-1.8941
Chenodeoxycholate	0.28667	-1.8025
Taurine	0.29017	-1.785
Hexanal	3.41740	1.7729
alpha-Glucosamine 1-phosphate	0.30506	-1.7128
LPA 20:1	0.30837	-1.6972
gamma-Butyrolactone	0.31093	-1.6854
Uric acid	0.31128	-1.6837
D-Turanose	2.86100	1.5165
d-Glycero-d-galacto-heptose	0.35560	-1.4917
Glycylglycine	0.36367	-1.4593
Glycohyocholic acid	2.72510	1.4463
DL-Tyrosine	0.38423	-1.3799
L-Threonine	2.46710	1.3028
PE 36:2	0.41087	-1.2833
3-Hydroxypropanoate	0.43139	-1.2129
Deoxyribose uracil	0.44820	-1.1578
Hydroxyproline	0.44833	-1.1574
O-Butanoylcarnitine	0.45392	-1.1395
3-Epicholic acid	0.45526	-1.1352
Diisopropanolamine	0.46100	-1.1172
Glyceryl monooleate	0.46157	-1.1154
Sulphuric acid	0.47219	-1.0826
1,2-Butanediol	0.47745	-1.0666
L-(+)-Arabinose	0.48270	-1.0508

**Table 8:** Differential metabolites.

Metabolite name	Fold Change	log2(FC)
Isophthalic acid	10.903	3.4466
D-Turanose	0.34598	-1.5312
Myo-Inositol	2.6251	1.3923
Glycohyocholic acid	0.39543	-1.3385
Valerolactam	0.41755	-1.26
(S)-Oleuropeic acid	0.43048	-1.216
FA 20:0	0.43671	-1.1953
LPE 20:2	0.44896	-1.1553
Lithocholic acid	2.1136	1.0797
Glycodeoxycholic acid	0.47848	-1.0635
Palmitoleic acid	2.0717	1.0508
Arginine	0.48483	-1.0444
Chenodeoxycholate	2.0283	1.0203

### Metabolomics Reveal Correlated Differential Metabolic Pathways Between Normal, OP-OA, and EXD

**Figure 6:**
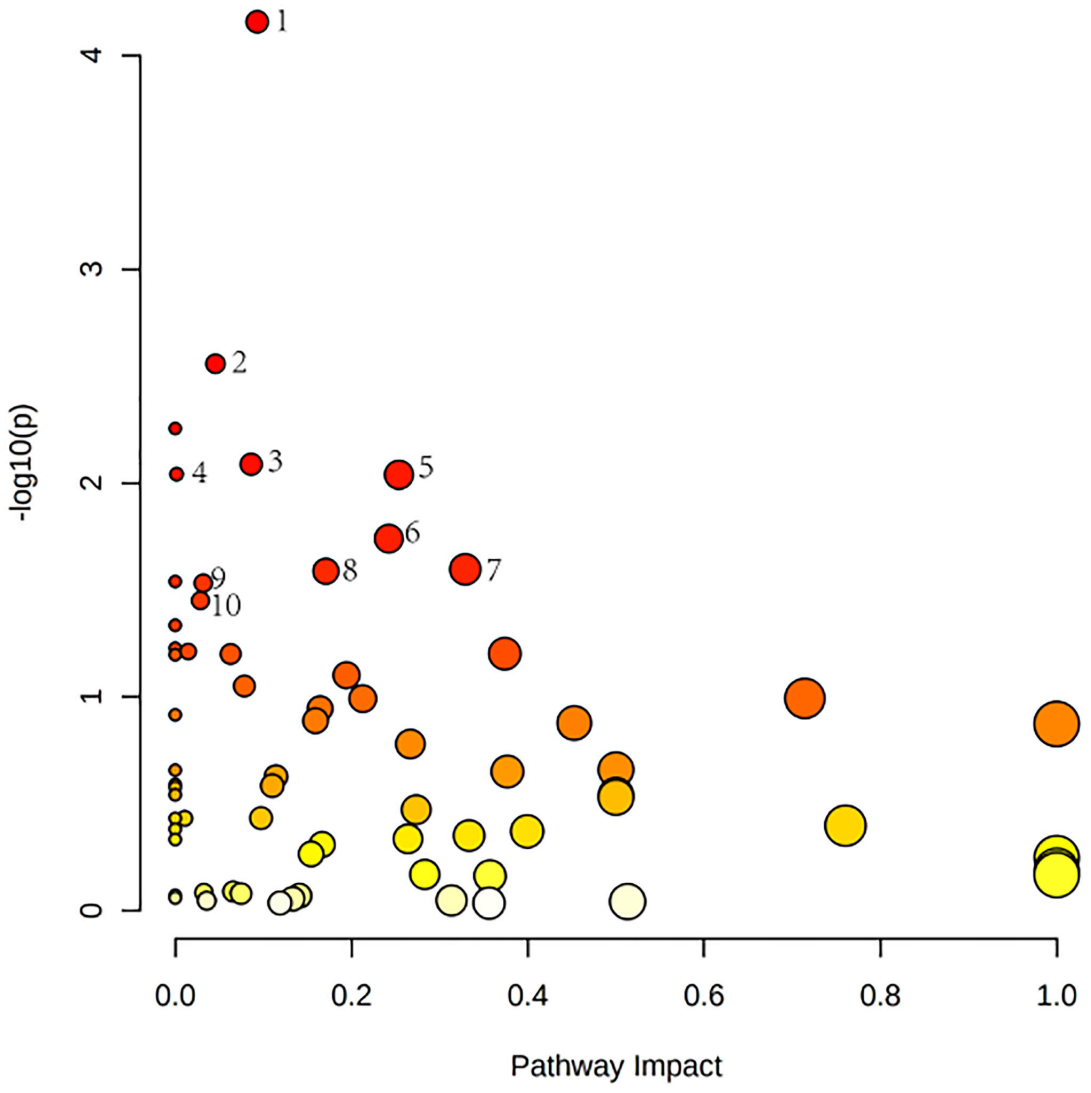
Metabolomics reveals correlated differential metabolic pathways between normal, OP-OA, and EXD. (A) Metaboanalyst (http://www.Metabo-analyst.ca/) carried out metabolic pathway enrichment analysis, with P<0.05, 10 related metabolic pathways were analyzed.

Relevant differential metabolites were imported into Metaboanalyst (http://www.Metabo-analyst.ca/) for metabolic pathway enrichment analysis, and a total of 10 metabolic pathways were analyzed with *P<0.05* (**Table 9**): Purine metabolism, phosphatidylinositol signaling system, glycolysis/gluconeogenesis, pyruvate metabolism, arginine biosynthesis, retinol metabolism, pyrimidine, pentose phosphate pathway, butanoate metabolism, pantothenate, and CoA biosynthesis (**Figure 6**). The comparison results showed that under the intervention of EXD, the most significant effect was purine metabolism.

**Table 9:** Pathway.

NO.	Pathway Name	Match Status	p	-log(p)	FDR	Impact	Details
1	Purine metabolism	9/65	6.97E-05	4.157	0.00467	0.09299	KEGG SMP
2	Phosphatidylinositol signaling system	2/28	0.00277	2.5578	0.09274	0.04561	KEGG
3	Glycolysis/Gluconeogenesis	5/26	0.00817	2.0879	0.10210	0.08612	KEGG SMP
4	Pyruvate metabolism	2/22	0.00909	2.0414	0.10210	0.00156	KEGG SMP
5	Arginine biosynthesis	8/14	0.00914	2.0389	0.10210	0.2538	KEGG
6	Retinol metabolism	1/17	0.01819	1.7402	0.17409	0.24227	KEGG SMP
7	Pyrimidine metabolism	10/39	0.02532	1.5966	0.17898	0.32899	KEGG SMP
8	Pentose phosphate pathway	2/22	0.02584	1.5876	0.17898	0.17086	KEGG SMP
9	Butanoate metabolism	4/15	0.02939	1.5319	0.17898	0.03175	KEGG SMP
10	Pantothenate and CoA biosynthesis	6/19	0.03545	1.4503	0.19795	0.02857	KEGG SMP

## Discussion

In this study, we used GC-TOFMS and LC-QTRAP-MS/MS techniques to study the metabolomic differences between OA and Osteoporotic OA, as well as the target and efficacy mechanism of EXD in Osteoporotic OA. Our study shows that the OP environment has a promoting effect on the pathological progression of OA, mainly manifested as the elevated gene and protein expressions of cartilage-related markers SOX9, COL2A1, and COMP and the aggravation of osteoporosis under the influence of bone metabolism, while EXD has a negative effect on Osteoporotic OA and has a significant intervention effect, which is mainly reflected in reducing the gene and protein expressions of cartilage-related markers SOX9, COL2A1, and COMP, thereby protecting cartilage, relieving osteoporosis, and improving bone and joint structure and environment (26–28).The intervention of chenodeoxycholate, D-Turanose, cystine and other metabolites may regulate and purine metabolism, phosphatidylinositol signaling system, glycolysis/gluconeogenesis, pyruvate metabolism, arginine biosynthesis, visual flavonol metabolism, pyrimidine metabolism, pentose phosphate pathway, butyrate metabolism, pantothenate, and CoA biosynthesis pathways are related.

EXD has a regulatory effect on the above metabolites. In order to further clarify the medicinal substances of EXD, we studied the composition of EXD. The middle peak is higher, which may be the main pharmacodynamic substance of EXD in the treatment of Osteoporotic OA. Combined with literature analysis, the findings are consistent with our research. Gentiopicroside, as one of the active ingredients of gentiana grandis, showed effective protection against IL-1β-induced inflammatory response of rat articular chondrocytes and alleviated the progression of OA (29). At the same time, studies have also shown that gentiopicroside can improve OP by regulating the β-catenin-bone morphogenetic protein 2 signaling pathway to promote the osteogenic differentiation of bone mesenchymal stem cells (30). Hydroxy anthraquinone compounds in a variety of herbal medicines have anti-inflammatory, antibacterial, antiviral, antitumor, antitussive, antihypertensive, and other pharmacological effects, and have broad research prospects. Hu et al. showed that emodin can protect rat knee cartilage through the anti-matrix degradation pathway, thereby alleviating the further development of OA (31). Kim JY et al. showed that emodin can also promote bone remodeling by regulating abnormal bone metabolism. Quercetin alleviates rat OA by inhibiting chondrocyte inflammation and apoptosis, regulating synovial macrophage polarization into M2 type macrophages, and can also regulate autophagy and apoptosis improvement in rat osteocytes and bone metabolism, thereby alleviating OP symptoms caused by oophorectomy (32). In addition, diosmetin, wogonin, and other monomers have also been confirmed in existing studies to play an anti-inflammatory and cartilage protective role in OA and OP by regulating related signaling pathways, inhibiting osteoclast differentiation, and activating intrachondral signaling pathways (33–36). It can be regarded as the active pharmaceutical ingredient of EXD.

Both GC-TOFMS and LC-QTRAP-MS/MS are techniques widely used for metabolite component analysis and related pathway identification with good agreement. These techniques have good potential for rapid analysis of non-targeted metabolite profiles and have great advantages in the study of metabolites and related pathways intervened by traditional Chinese medicine compounds. Hossam M Abdallah et al. used GC-TOFMS and LC-QTRAP-MS/MS to study the anti-osteoporosis and bone protection effects of ovariectomized rats under the intervention of Lepidium sativum extract (37). A Batushansky et al. used the GC-TOFMS method to study OA, showing the characteristics of articular cartilage metabolism and the plasticity of chondrocyte metabolism, providing a basis for further research on the role of cartilage metabolism in OA (38). In our study, the differential metabolites of normal compared with OA were found mainly by metabolomic identification: PE 36:2, N-Amidino-L-aspartate, LPA 20:1, Lactulose, D-Mannitol, taurine, D-Turanose, (S)-Oleuropeic acid, 3-Hydroxypropanoate, cystine, and the differential metabolites between the normal group and the OP-OA group are isophthalic acid, cystine, chenodeoxycholate, taurine, hexanal, LPA 20:1, uric acid, D-Turanose, glycylglycine, glycohyocholic acid and other 27. By comparison, it was found that D-Turanose, cystine, and chenodeoxycholate may be the key factors for OP to accelerate the progression of OA.

From our study, we found that the metabolites D-Turanose, cystine, and chenodeoxycholate were most significantly different in the OP-OA group and the OA group. D-Turanose is a carbohydrate metabolite, and the study by Yan Chao Cui et al. showed that the growth of osteoclasts depends on glycolysis as the main energy-producing pathway involved in the regulation of bone metabolism and has a role in cartilage and bone repair, thereby delaying OP progression (39). Cystine, as an oxidized dimer compound of cysteine, is a frequently occurring form of cysteine in organic tissues and is important to the progression of OA. Wei-Yuan Tsaiet et al. showed that glutathione is synthesized in chondrocytes to induce cartilage destruction through the cystine/glutamate anti-transport system, and inhibition of this system delays further cartilage destruction (40). Chenodeoxycholate is the main active component of animal bile. Zhao et al. demonstrated that chenodeoxycholate can effectively alleviate cartilage degeneration on the surface of femoral condyle in rabbit OA model, reduce articular cartilage damage, joint erosion, and the expression of related factors, thereby reducing pathological changes in articular cartilage and synovium, delaying the progression of OA (41). The above metabolites and related pathways were found to be consistent with our research predictions through literature search.

To sum up, this study found that there are many metabolic differences between Osteoporotic OA and OA. Among them, cystine and chenodeoxycholate may be the key to OP aggravating the key damage of OA. EXD has good effects on Osteoporotic OA. Emodin, quercetin, and gentiopicroside may be its main active substances. In addition, the intervention effect of EXD on Osteoporotic OA may be related to the metabolic differences of D-Turanose and chenodeoxycholate, involving metabolic pathways such as glycolysis/gluconeogenesis, pantothenate, and CoA biosynthesis. Our study elucidates the metabolic factors that promote the progression of OA for OP and provides evidence for the target and efficacy mechanism of EXD in the treatment of Osteoporotic OA.

## Data Availability Statement

The data reported in this paper have been deposited in the OMIX, China National Center for Bioinformation / Beijing Institute of Genomics, Chinese Academy of Sciences (https://ngdc.cncb.ac.cn/omix: accession no. OMIX001328).

## Ethics Statement

The animal study was reviewed and approved by the Ethics Committee of the Affiliated Hospital of Nanjing University of Chinese Medicine.

## Author Contributions

ZM, YW, and PW participated in the design of the study and wrote the manuscript. LZ, XS, and RX analyzed the data. XL, TL, NY, and LJ performed the experiments. All authors read and approved the final manuscript.

## Funding

This work was supported by the Peak Academic Talent Project of Jiangsu Province Hospital of Chinese Medicine (y2021rc02, y2021rc20) and the General Project of Natural Fund of Nanjing University of Traditional Chinese Medicine (k2021x05).

## Conflict of Interest

The authors declare that the research was conducted in the absence of any commercial or financial relationships that could be construed as a potential conflict of interest.

## Publisher’s Note

All claims expressed in this article are solely those of the authors and do not necessarily represent those of their affiliated organizations, or those of the publisher, the editors and the reviewers. Any product that may be evaluated in this article, or claim that may be made by its manufacturer, is not guaranteed or endorsed by the publisher.
